# Effectiveness of the integrated care model *Salut+Social* in patients with chronic conditions: A mixed methods study protocol

**DOI:** 10.1097/MD.0000000000019994

**Published:** 2020-05-08

**Authors:** Ester Gavaldà-Espelta, Maria del Mar Lleixà-Fortuño, Jordi Baucells-Lluis, Maria Ferré-Ferraté, Gerard Mora-López, Begoña Tomàs-Navarro, Claudia Curto-Romeu, Jorgina Lucas-Noll, Carina Aguilar Martin, Alessandra Queiroga Gonçalves, Carmen Ferré-Grau

**Affiliations:** a Direcció d’Atenció Primària Terres de l’Ebre, Gerència Territorial Terres de l’Ebre, Institut Català de la Salut, Tortosa; b Departament d’Infermeria, Programa de Doctorat Infermeria i Salut, Universitat Rovira i Virgili, Tarragona; c Direcció de Serveis Territorials de Salut a les Terres de l’Ebre, CatSalut, Generalitat de Catalunya, Tortosa, Spain; d Direcció de Sistemes d’Informació i Comunicació, Gerència Territorial Terres de l’Ebre, Institut Català de la Salut, Tortosa; e Equip d’Atenció Primària Amposta, Gerència Territorial Terres de l’Ebre, Institut Català de la Salut, Tortosa; f Unitat d’Avaluació, Direcció d’Atenció Primària Terres de l’Ebre, Institut Català de la Salut, Tortosa; g Unitat de Suport a la Recerca Terres de l’Ebre, Fundació Institut Universitari per a la recerca a l’Atenció Primària de Salut Jordi Gol i Gurina (IDIAPJGol), Tortosa; h Unitat Docent de Medicina de Família i Comunitària Tortosa-Terres de L’Ebre, Institut Català de la Salut, Tortosa, Tarragona, Spain.

**Keywords:** chronic disease, implementation science, information technology, integrated health care systems, primary health care, qualitative research, quality of life

## Abstract

**Introduction:**

Integrated care models aim to provide solutions to fragmentation of care by improving coordination. This study will evaluate the effectiveness of a new integrated care model (*Salut* *+* *Social*), which will promote the coordination and communication between social and healthcare services in southern Catalonia (Spain) to improve quality of life, adherence to treatment and access to medical services for patients with chronic conditions, and also to reduce caregiver burden. Additionally, we will evaluate the experience of caregivers, health professionals and social workers with the new model implemented.

**Methods and analysis:**

A clinical trial using mixed methodology will be carried out. The intervention consists of improving the coordination between the social and healthcare sectors during a 6-month period, by means of information and communication technology (ICT) tools that operate as an interface for the integrated care model. The study subjects are primary care patients with chronic health and social conditions that can benefit from a collaborative and coordinated approach. A sample size of 141 patients was estimated. Questionnaires that assess quality of life, treatment adherence, medical service and caregiver burden will be used at baseline and at 6, 9, and 12 months after the beginning of the study. The principal variable is quality of life. For statistical analysis, comparisons of means and proportions at different time points will be performed. A discussion group and semi-structured interviews will be conducted with the aim of improving the care model taking into account the opinions of professionals and caregivers. A thematic content analysis will be carried out.

**Ethics and dissemination:**

This study protocol has been approved by the Clinical Research Ethics Committee of the Fundació Institut Universitari per a la Recerca a l’Atenció Primària de Salut Jordi Gol i Gurina (code P17/100). Articles will be published in international, peer-reviewed scientific journals.

**Trial registration:**

Clinical-Trials.gov: NCT04164160.

## 1 Introduction

One of the most significant demographic trends globally is the ageing of the population, with chronicity and multimorbidity increasing in high income countries. It is estimated that the percentage of the world's population older than 60 years will increase from 12% to 22% between 2015 and 2050.^[1]^ Health and social systems need to take the necessary steps to prepare for this demographic change in order to ensure adequate care.^[2]^

Chronic diseases are characterized by a long-term progression, limiting the quality of life of patients and caregivers, causing premature mortality and financial burden to families and society.^[3]^ Crucially, adults with multiple chronic conditions are the main users of healthcare services in all age ranges, and they account for two-thirds of healthcare spending. Consequently, increasing effectiveness of care in this group of patients should become a priority.^[4]^

Despite the differences between countries regarding the care of patients with chronic conditions, they all share similar challenges, including fragmented services, inefficient systems, poor quality, and spiralling costs.^[5]^ In recent years, new conceptual frameworks for the care of patients with chronic conditions have been developed, based on successful interventions such as the Chronic Care Model, which has achieved wide acceptance and dissemination and has inspired other models such as the Innovative Care for Chronic Conditions (ICCC)^[5]^ and the Expanded CCM.^[6,7]^

Integrated and person-centred care is a model increasingly incorporated into policies^[8,9]^ aimed to improve access, quality, continuity, effectiveness, and sustainability of healthcare systems.^[10]^ According to Shaw et al,^[11]^ integrated care is an organising principle for healthcare delivery that aims to optimize patient care by means of intensifying coordination between services. In this model, a collaborative and shared approach responds to patients’ needs.

The integration of social and healthcare services remains a challenge for healthcare systems in Europe and globally. A systematic review that analyzed the effectiveness of integrated care interventions in improving the quality of life for patients with chronic conditions concluded that Chronic Care Model interventions were beneficial, although the effectiveness increased with the number of components.^[12]^

Importantly, new healthcare technology is rapidly expanding, offering new opportunities for information sharing among departments and institutions and facilitating patients’ access to services.^[13–15]^

The principal objective of this study is to evaluate the effectiveness of a new integrated care model developed with the support of an information and communication technology (ICT) tool (named *Salut* *+* *Social*) in patients with chronic conditions, regarding quality of life and treatment adherence, medical service utilization and caregiver burden. The secondary objective is to analyse opinions and experiences of health and social professionals and caregivers with the new model implemented. The combination of quantitative and qualitative methodology, as established by the Medical Research Council,^[16–18]^ will provide a better evaluation of the intervention since it considers participants’ needs and environmental characteristics that might influence acceptability, adaptability, adherence, feasibility, and compatibility.

## 2 Methods and design

**Figure 1:**
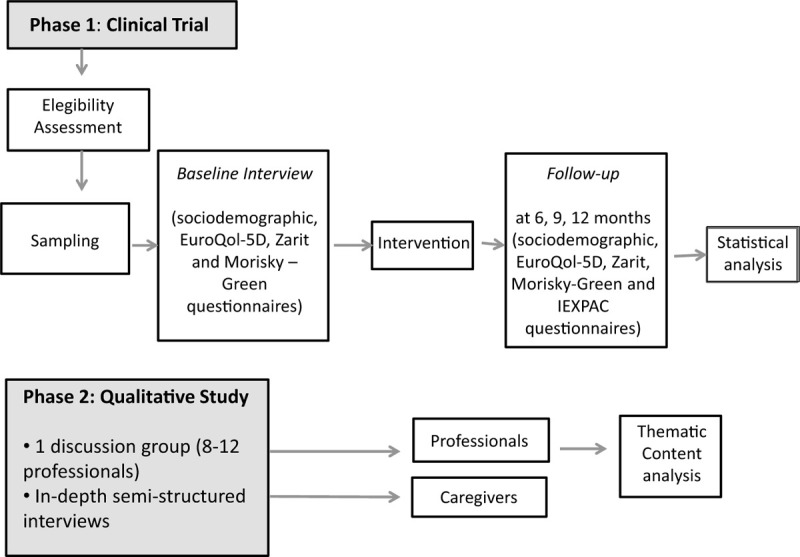
Study flowchart.

A mixed methods study of 2 phases, following Medical Research Council guidelines^[16]^ on the evaluation of complex interventions, will be conducted between June 2019 and June 2020. Figure 1 shows the study flowchart.

### 2.1 Setting and participants

Participants of the study are patients with chronic conditions and their informal caregivers from two primary care centres (PCC) of the *Institut Català de la Salut* (ICS) (*Ribera d’Ebre, Gerència Territorial Terres de l’Ebre, Catalunya*) that meet the health and social inclusion criteria.

#### 2.1.1 Inclusion criteria (all criteria must be met)

- Adult patients with at least one health and one social condition, as specified below:
  (a) Health condition, as registered in the electronic medical records of the ICS: complex chronic patient; advanced chronic disease; patient in the home care programme; dementia; neurodegenerative disease; stroke; other chronic diseases.
  (b) Social condition, according to the social services workstation from the *Ribera d’Ebre* district: dependence; home social services assistance; teleassistance systems assigned by the home support services.
- Knowledge of Spanish or Catalan
- Accepting participation in the study (informed consent signed by the patient or the caregiver).

#### 2.1.2 Exclusion criteria

- Institutionalized patients.
- Users with difficulties filling out or responding to questionnaires.

### 2.2 Phase 1: effectiveness of the intervention

#### 2.2.1 Study design and sample size

We will carry out a single group clinical trial to evaluate the effect of the implementation of a new integrated care model. The principal dependent variable is quality of life. In order to detect a difference of at least 3 units in the EuroQol-5D questionnaire, with 95% confidence and a power of 80%, the sample size should be at least of 141 subjects. Convenience sampling will be used.

#### 2.2.2 Intervention

The intervention consists of promoting the coordination between the social and health sectors during a 6-month period by means of ICT tools that create an interface for the “*Salut* *+* *Social”* model, a new integrated care model. The ICT tools consist of a web application (app) and a mobile multicentre and multirole app, which maintains a constant communication flow between professionals from the different fields (primary healthcare, social services and hospitals), in order to share patients’ information and coordinate the actions that need to be implemented. The app also includes a suggestion box that healthcare and social services professionals can use to send suggestions for technical improvement, propose changes of processes and notify of app errors.

Nurses, general practitioners and social workers will participate in the intervention. Due to the lack of social workers in the primary care services of the study area, municipal social workers will be invited to participate in the study and will have assigned patients. Health professionals and social workers will inform users and their caregivers of available resources and grants, information on basic social services and on the Dependence Law.

The app should detect if the person requires social resources and will help allocate the necessary resource more effectively by joint action.

#### 2.2.3 Training of health professionals and social workers

Before starting the study, participating professionals (nurses, general practitioners, and social workers) will receive training on aspects related to the use of the Salut + Social app, and how to access it from the webpage and the mobile app. A video with instructions will be posted in a YouTube channel.

#### 2.2.4 Recruitment and data collection

Patients who meet the inclusion criteria will be selected by professionals and will be signed up to the app. In this step, clinical and social data of the patients will be automatically entered in the app and the professionals will propose coordinated actions with each patient. Next, patient and/or caregiver will attend a first interview with the nurse in charge of this task.

In the first interview, patients or caregivers will be asked to respond to the study questionnaires (ad hoc questionnaire for sociodemographic data, EuroQol-5D,^[19,20]^ Zarit questionnaire^[21,22]^ and Morisky-Green test^[23,24]^) and will participate in future care planning. If the nurse estimates that insufficient information for adequate integrated care exists, a joint visit with health professionals and social workers that can take place either in the health centre or at home could be requested through the app. Joint visits may be requested at any stage of the study whenever considered necessary.

Patients will receive an appointment to attend their PCC at 6, 9, and 12 months after their incorporation into the programme. During follow-up visits, patients or caregivers will be asked to respond to the same questionnaires, together with the IEXPAC questionnaire,^[25]^ which evaluates the experience of the chronic patient with the new care model.

Changes in the health and social needs of the patients and new interventions performed by any professional during the study period will be communicated through the app, enabling the coordination of all professionals involved.

#### 2.2.5 Outcomes and measurement instruments

The main result variable of the study is quality of life (EuroQol-5D). Table 1 shows the study variables, the measurement instruments and the timing of data collection.

**Table 1:**
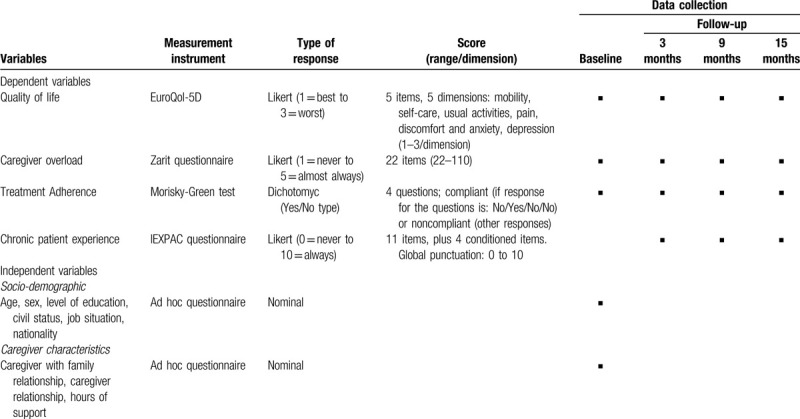
Study variables, measurement instruments and timing of data collection.

#### 2.2.6 Statistical analysis

A descriptive analysis of all investigated variables will be provided, using means and standard deviations for quantitative variables and percentages with their confidence intervals for qualitative variables. We will evaluate if there is significant improvement in quality of life and a decrease in caregiver burden through a test of paired means, as well as improvement in treatment adherence using McNemar test of paired proportions before and after the intervention (after 6 months). Statistical significance is established at *P* ≤ .05, with a 95% confidence level.

### 2.3 Phase 2: qualitative evaluation of the intervention

The aim of this phase is to detect improvable aspects of the intervention according to the opinion and experiences of caregivers, health professionals, and social workers.

#### 2.3.1 Study design

Interpretive, qualitative research.

#### 2.3.2 Participants, sampling, and recruitment

We will include the professionals and caregivers that participate in the intervention. Professionals of different gender, age, primary care centre and disciplines (nurse, general practitioners, and social workers) will be invited in order to achieve maximum discursive variability. Also, caregivers from patients of different genders, age, socioeconomic situation, and conditions (health and social) will be selected.

#### 2.3.3 Data collection

Twelve months after the beginning of the *Salut* *+* *Social* programme, a discussion group will be conducted in one of the PCC of the study. The discussion group will consist of 8 to 12 professionals, will have 1-h duration and will follow a topic list previously designed by the research team. If discourse saturation is not achieved with the planned discussion group, we will organize an additional discussion group of similar characteristics.

The discussion group will be audio recorded and literally transcribed with the consent of all participants. If all participants agree, it will be video recorded. In order to guarantee confidentiality, the identifying data of the participants will be anonymized in the transcripts.

In-depth semi-structured interviews will be conducted with the caregivers, until reaching data saturation.

#### 2.3.4 Data analysis

For the analysis of qualitative data, a corpus text composed of field notes and the transcripts will be analysed by at least three members of the research team. The corpus text will be read successively and pre analytical intuitions will be formulated. In meetings with the research team, a thematic interpretive content analysis will be performed and then triangulated. The Atlas-Ti programme will be used to support the analysis if necessary.

We will perform an interpretation of the meanings, and an explanatory framework will be created with the contributions of each type of informant (professionals and caregivers participating in the study).^[26]^

### 2.4 Ethics and dissemination

The study protocol (version 1, 13/11/2019) has been approved by the Clinical Research Ethics Committee of the *Fundació Institut Universitari per a la Recerca a l’Atenció Primària de Salut Jordi Gol i Gurina* (IDIAPJGol) (code P17/100). This study will follow the tenets of the Declaration of Helsinki and Good Clinical Research Practice, with emphasis on the right to privacy, anonymity and confidentiality. Patients and caregivers will be provided with a fact sheet of the study and will sign the informed consent form. In case of amendments of the study protocol, a new version will be submitted to the Ethics Committee for approval and the clinical trials registry will be updated.

Participants in the focus groups and interviews will sign the authorization for recording sessions. All the instruments are inside the app for data protection purposes. A username and password are required to access the app. The Primary Care Management from the *Terres de l’Ebre* supports the implementation of the app and approves the project.

The results of the study will be firstly communicated to participating patients, caregivers, and professionals by means of local meetings. For the dissemination to the general population, we will schedule an informative meeting and the local and national media will be contacted. Finally, the results of the study will be presented in national and international meetings and will be published in scientific journals.

## 3 Discussion

This is an innovative project in Catalonia, which aims to evaluate the effectiveness of a new integrated care model that takes into account the health and social conditions of patients with chronic conditions using ICT support. The project intends to strengthen the role of primary care services as promoter of integrative care for patients with chronic conditions.

This project is inspired by the current health policy published in the Health Plan of Catalonia 2016 to 2020, which recommends the collaboration between services and professionals, and suggests the transformation of the health system toward a more integral and person-centred care model. The Plan also promotes the use of ICT to facilitate the collaboration between institutions, care services and professionals, in order to provide better healthcare, avoid redundancies, and optimize resources.^[27]^

The study intends to respond to problems that arise from deficits in communication and coordination between social and health systems, such as the lack of a strategy regarding joint evaluation of patients, which generates duplication of information and slows down delivery of care. Since in our region the PCC do not have social workers, the health professionals need to contact municipal social workers to promote integration of care, underscoring the need for improvement in communication in this geographical area.

Because this study will be conducted in a small geographical area of southern Catalonia, the results obtained might not be replicable. However, we believe that our results will be of interest to other regions of Spain and also to the international community, since to date studies that evaluate the implementation of new models for integrating social and healthcare are scarce.^[28–30]^ Another limitation of the study might be the loss of participants due to worsening health and even death.

The qualitative phase of the study will offer data on feasibility, acceptability, barriers, and facilitators of the intervention, based on the opinion of participating professionals and caregivers. The data obtained in this phase will be useful to adapt and improve the integrated care model according to participants’ experience. In a study that evaluated nine models of integrated community-based primary healthcare, authors identified barriers to a more innovative use of technology that was linked to information access, limited functionality of technology and organizational and provider inertia.^[13]^

## Acknowledgments

This work has been carried out within the framework of the Programa *de Doctorat Infermeria i Salut* from the *Universitat Rovira i Virgili.*

The authors thank the following professionals who are participating on research support and data collection of the study: Miriam Moira-Costa (Equip d’Atenció Primària (EAP)Flix, ICS), Armand Pi-Coll (EAP Flix, ICS), Rosa Amor-Gonzalez (EAP Flix, ICS), Laia Sabaté-Arnau (EAP Mora la Nova-Mora d’Ebre, ICS), Montserrat Escribà Aguilà (EAP Mora la Nova-Mora d’Ebre, ICS), Anna Rel-Puyo (Consell Comarcal de serveis socials de Ribera d’Ebre), José Fernandez Sáez (Unitat de Suport a la Recerca Terres de l’Ebre, IDIAPJGol, Tortosa), Nuria Brunet Reverté (EAP Amposta, ICS), Maria Teresa Irigoyen Garcia (EAP Amposta, ICS), Mercè Princep Guarch (Consell Comarcal de serveis socials de Amposta), Assumpta Eixarch Conesa (Consell Comarcal de serveis socials de Amposta) and Anna Porta (Consell Comarcal de serveis socials de Amposta).

## Author contributions

**Conceptualization:** Ester Gavaldà-Espelta, Maria del Mar Lleixà-Fortuño, Jordi Baucells-Lluis, Maria Ferré-Ferraté, Carmen Ferré-Grau.

**Data curation:** Ester Gavaldà-Espelta, Maria del Mar Lleixà-Fortuño, Jordi Baucells-Lluis, Maria Ferré-Ferraté, Gerard Mora-López, Begoña Tomàs-Navarro, Claudia Curto-Romeu, Jorgina Lucas-Noll, Carmen Ferré-Grau.

**Formal analysis:** Gerard Mora-López, Begoña Tomàs-Navarro, Carina Aguilar Martín, Alessandra Queiroga Gonçalves.

**Funding acquisition:** Ester Gavaldà-Espelta, Maria del Mar Lleixà-Fortuño, Jordi Baucells-Lluis, Maria Ferré-Ferraté, Carmen Ferré-Grau.

**Investigation:** Ester Gavaldà-Espelta, Maria del Mar Lleixà-Fortuño, Jordi Baucells-Lluis, Maria Ferré-Ferraté, Gerard Mora-López, Begoña Tomàs-Navarro, Claudia Curto-Romeu, Jorgina Lucas-Noll, Carmen Ferré-Grau.

**Methodology:** Ester Gavaldà-Espelta, Carina Aguilar Martín, Alessandra Queiroga Gonçalves, Carmen Ferré-Grau.

**Project administration:** Ester Gavaldà-Espelta, Maria del Mar Lleixà-Fortuño, Begoña Tomàs-Navarro, Carmen Ferré-Grau.

**Supervision:** Ester Gavaldà-Espelta, Maria del Mar Lleixà-Fortuño, Gerard Mora-López, Carmen Ferré-Grau.

**Validation:** Gerard Mora-López.

**Visualization:** Gerard Mora-López.

**Writing – original draft:** Ester Gavaldà-Espelta, Maria del Mar Lleixà-Fortuño, Jordi Baucells-Lluis, Maria Ferré-Ferraté, Gerard Mora-López, Begoña Tomàs-Navarro, Claudia Curto-Romeu, Jorgina Lucas-Noll, Carina Aguilar Martín, Alessandra Queiroga Gonçalves, Carmen Ferré-Grau.

**Writing – review & editing:** Ester Gavaldà-Espelta, Maria del Mar Lleixà-Fortuño, Jordi Baucells-Lluis, Maria Ferré-Ferraté, Gerard Mora-López, Begoña Tomàs-Navarro, Claudia Curto-Romeu, Jorgina Lucas-Noll, Carina Aguilar Martín, Alessandra Queiroga Gonçalves, Carmen Ferré-Grau.
